# Isolation and purification of Tartary buckwheat polysaccharides and their effect on gut microbiota

**DOI:** 10.1002/fsn3.3072

**Published:** 2022-09-27

**Authors:** Yue Xiang, Ya‐Nan Cao, Si‐Hui Yang, Yuan‐Hang Ren, Gang Zhao, Qiang Li, Hehe Li, Lian‐Xin Peng

**Affiliations:** ^1^ Key Laboratory of Coarse Cereal Processing of Ministry of Agriculture and Rural Affairs, Sichuan Province Engineering Technology Research Center of Coarse Cereal Industrialization Chengdu University Chengdu People's Republic of China; ^2^ Key Laboratory of Brewing Molecular Engineering of China Light Industry Beijing Technology and Business University Beijing People's Republic of China

**Keywords:** gut microbiota, probiotics, short‐chain fatty acids, Tartary buckwheat polysaccharides

## Abstract

Tartary buckwheat (*Fagopyrum tataricum*) is rich in polysaccharides that can be utilized by the gut microbiota (GM) and provide several health benefits. However, the mechanisms underlying the action of these polysaccharides remain unclear to date. In this study, Tartary buckwheat polysaccharides (TBP) were purified, and five fractions were obtained. The composition of these fractions was determined using ion chromatography. Different TBP components were investigated regarding their probiotic effect on three species of *Bifidobacteria* and *Lactobacillus rhamnosus*. In addition, the effect of TBP on GM and short‐chain fatty acids (SCFAs) was evaluated. Results showed that the probiotic effect of TBP fraction was dependent on their composition. The polysaccharides present in different fractions had specific probiotic effects. TBP‐1.0, mainly composed of fucose, glucose, and d‐galactose, exhibited the strongest proliferation effect on *L. rhamnosus*, while TBP‐W, rich in glucose, d‐galactose, and fructose, had the best promoting effect on *Bifidobacterium longum* and *Bifidobacterium adolescentis* growth. Furthermore, TBP‐0.2, composed of d‐galacturonic acid, d‐galactose, xylose, and arabinose, exhibited its highest impact on *Bifidobacterium breve* growth. The composition of GM was significantly altered by adding TBP during fecal fermentation, with an increased relative abundance of *Lactococcus*, *Phascolarctobacterium*, *Bacteroidetes*, and *Shigella*. Simultaneously, the level of SCFA was also significantly increased by TBP. Our findings indicate that Tartary buckwheat can provide specific dietary polysaccharide sources to modulate and maintain GM diversity. They provide a basis for Tartary buckwheat commercial utilization for GM modulation.

## Graphical abstract

The Tartary buckwheat polysaccharides present in different fractions had specific probiotic effects. The composition of gut microbiota was significantly altered by adding Tartary buckwheat polysaccharides during fecal fermentation. Tartary buckwheat can provide specific dietary polysaccharide sources to modulate and maintain gut microbiota diversity.

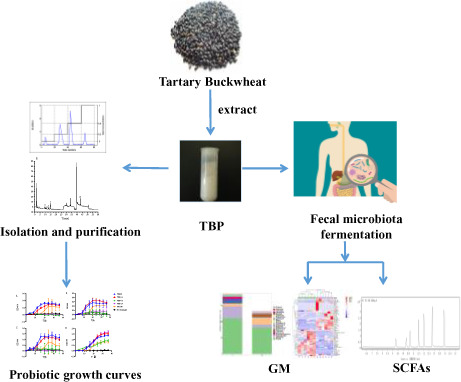

## 1 INTRODUCTION

Colonization of the gut microbiota (GM) in humans begins at birth and develops majorly in the first 3 years of life. It becomes more complex when children start eating solid food, gradually possessing microbiota roughly similar in structure and function to adults (Roswall et al., 2021). The GM composition in healthy adults is generally stable. However, when it is disturbed due to internal or external perturbations, the body often starts showing signs of various diseases. For instance, imbalanced GM in patients with type 2 diabetes decreased the number of common butyrate‐producing bacteria, resulting in increased growth of various opportunistic pathogens (Gilbert et al., 2018; Qin et al., 2012).

Polysaccharides, as an essential component of the daily diet, can be involved in various physiological activities through the regulation of GM (Seedorf et al., 2014). Polysaccharides exert significant modulatory effects on host health by promoting the growth of certain probiotic bacteria, such as *Lactobacilli* and *Bifidobacteria* (Fernández et al., 2016), and inducing the expression of immunomodulatory and pathogen antagonistic molecules (Turroni et al., 2014). Polysaccharides also promote the production of various types of short‐chain fatty acids (SCFAs) that play a crucial role in host metabolism (Krautkramer et al., 2021). It was found that after 24 h of anaerobic fermentation of *Porphyra haitanensis* polysaccharides (PHP), the GM composition was remodeled due to the proliferation of probiotics and the inhibition of pathogens. The level of GM diversity was also significantly increased. In addition, the final concentrations of acetic acid, propionic acid, butyric acid, and total SCFAs were increased (Xu et al., 2019). Recently, polysaccharides' physiological functions were found to be closely related to their structure in terms of monosaccharide composition, relative molecular mass, molecular shape, and chain conformation (Yang et al., 2022). It is also known that polysaccharides from different plant sources provide a rich and diverse carbon source for the GM and are crucial components in maintaining intestinal homeostasis (Tannock, 2020). Moreover, understanding the relationship between plant polysaccharides composition and the regulation of microbial activity provides a basis for adopting a precise diet in routine.

Tartary buckwheat (*Fagopyrum tataricum*), a dicotyledonous plant belonging to the genus Polygonaceae in Fagopyrum Mill (*Fagopyrum*), is mainly cultivated in the provinces in the south of the Yangtze River in China, including Sichuan, Guizhou, and Shanxi (Luthar et al., 2021). Tartary buckwheat products, such as tea and noodles, are popular among consumers. It is a plant with both medicinal and edible properties. It has many health benefits, including antioxidant, anti‐inflammatory, and antidiabetic effects which are closely related to its bioactive substances. A recent study has shown the α‐d‐glucosidase inhibitory and antidiabetic activity of Tartary buckwheat polysaccharide (TBP) (Zou et al., 2021). Although few studies have documented TBP separation, purification, and structural identification (Wang et al., 2016), the dissection of different TBP components, which may exert the probiotic effect and regulate the GM composition, is not reported to date.

In the current study, different polysaccharide fractions were extracted from Tartary buckwheat and purified further to investigate the effect of different TBP fractions on the proliferation of various probiotics (*Bifidobacterium* and LGG). The regulation of GM and their metabolites composition by different TBP fractions was also elucidated. Results showed that different TBP components have different prebiotic effects on various probiotics. TBP has a regulatory effect on the composition of GM and the production of SCFAs. These findings will clarify the physiological activities of Tartary buckwheat and provide a theoretical reference for the commercial development and utilization of Tartary buckwheat.

## 2 MATERIALS AND METHODS

### 2.1 Plant materials and bacterial strains

Tartary buckwheat (Chuanqiao No. 1) was provided by the Key Laboratory of Miscellaneous Grains Processing, Ministry of Agriculture and Rural Affairs, Chengdu University. The lyophilized powder of bacterial strains, *Bifidobacterium longum* (ATCC 15707), *Bifidobacterium breve* (ATCC 15700), *Bifidobacterium adolescentis* (ATCC 15703), and *Lactobacillus rhamnosus* (ATCC 53103) was procured from Mingzhou Biotechnology Co., Ltd.

### 2.2 Extraction of TBP

The Tartary buckwheat was washed and dried. Afterward, 10 times the volume of distilled water was added. The mixture was subjected to boiling water extraction for 3 h, which was repeated three times. The impurities of the supernatant liquor were removed using a filter. The extracts were then combined and concentrated. Protein was removed using the Sevag reagent (chloroform/1‐Butanol, v/v = 4:1) followed by centrifugation, and anhydrous ethanol (4‐volume) was added. The resulting solution was stirred and incubated overnight for protein precipitation. The precipitate was obtained by filtration using the Buchner funnel. Furthermore, the precipitate was redissolved in distilled water (60°C water bath to evaporate the ethanol). The ethanol‐free precipitate was subjected to lyophilization using a freeze‐drying machine (FD‐2, BoYikang Experimental Instrument Co., Ltd.) to obtain TBP.

### 2.3 Isolation and purification of TBP

The TBP was dissolved in the distilled water and centrifuged. The supernatant was used for the subsequent experiment. The four elution solvents used in this study were water, 0.2 M NaCl, 0.5 M NaCl, and 1.0 M NaCl. The phenol–sulfuric acid method was adopted and the scatter plot was constructed (Wu et al., 2020). Based on the peak shape, the results were collected respectively. After concentrating the relevant components, dialysis using 3500 Da dialysis bags (Sigma Chemical Company) and freeze‐drying were done. The highest content fraction was weighed, dissolved in the mobile phase, and then centrifuged. The resulting supernatant was further purified using a GPC Autopurifier system (BRT‐GS) procured from the Bo Rui Saccharide Biotech Co., Ltd. and collected by the online detection combined with a refractive index detector (RI‐502, SHODEX) to collect the symmetric peaks. The collection solution was concentrated using a rotary evaporator and freeze‐dried.

### 2.4 TBP structure identification

The molecular weight was determined using high‐performance gel permeation chromatography (HPGPC). The samples and standard solution were prepared and filtered through a 0.22‐μm microporous membrane. Subsequently, the solution was transferred to the injection vial. The chromatographic conditions were adjusted as follows: chromatographic column BRT105‐104‐102 was in tandem with gel column (8 × 300 mm), the mobile phase was 0.05 M sodium chloride solution, the flow rate was 0.6 ml/min, the column temperature was 40°C, the injection volume was 20 μl, and the detector was a RI‐10A of the refractive index detector.

Monosaccharide composition was determined using an ion chromatography (IC). The solution of each monosaccharide was prepared as a 5 mg/L mixed standard. The sample was weighed in an ampoule, 3 M trifluoroacetic acid (TFA) was added, and hydrolyzed at 120°C for 3 h. The acid hydrolyzed solution was transferred to a tube and freeze‐dried with liquid nitrogen. Then, 5 ml of water was added to it and vortexed. In a separate tube, 100 μl of the suspension was aspirated and 900 μl of deionized water was added followed by centrifugation. The resulting supernatant was then analyzed using IC. The chromatographic conditions were adjusted as follows: column Dionex Carbopac™ PA20 (3 × 150); mobile phase A: H_2_O; B: 15 mM sodium hydroxide; C: 15 mM sodium hydroxide and 100 mM sodium acetate; flow rate: 0.3 ml/min; injection volume: 5 μl; column temperature: 30°C; and detector: electrochemical detect.

### 2.5 Effect of different fractions of polysaccharide on the growth of probiotics

Three *Bifidobacteria* (*B. longum*, *B. breve*, and *B. adolescentis*) and LGG were incubated in the lactic acid bacteria culture (MRS) medium for 2–3 days at 37°C for activation under anaerobic or aerobic conditions, respectively. The incubation was followed by three repeated passages before being prepared for use. The growth of the bacterial strains on different carbon source‐containing media was measured with an enzyme‐labeled instrument (Salli et al., 2021). Briefly, 20 μl of each polysaccharide fraction (2%) or glucose (2%) solution was added to the wells of the enzyme‐linked immunosorbent assay (ELISA) plate, followed by the addition of 180 μl of cell suspension containing the microorganism to be measured (1%, v/v). The final concentration of carbon source in each well was kept as 0.2% (w/v). Glucose was used as a nonselective positive control substrate. In addition, a medium without any added carbohydrate was used as a negative control. *Bifidobacterium* and LGG were incubated for 36 h at 37°C under anaerobic and aerobic conditions, respectively, and optical density (OD) was measured at 600 nm for every 4 h. The plates were shaken for 10 s before the measurements. Each bacterial–carbohydrate combination was analyzed in at least two independent experiments, each in three replicates.

### 2.6 Effect of TBP on fecal microorganisms

The medium preparation and fecal microorganism collection were performed based on the method described by Li et al. (2020). The fermentation medium contained of 2.0 g/L peptone, 2.0 g/L yeast powder, 0.1 g/L sodium chloride, 0.04 g/L potassium dihydrogen phosphate, 0.04 g/L dipotassium hydrogen phosphate, 0.01 g/L magnesium sulfate heptahydrate, 0.01 g/L calcium chloride hexahydrate, 2 g/L sodium bicarbonate, 0.02 g/L hemin, 0.5 g/L l‐cysteine 0.5 g/L bile salt, 2.0 ml/L Tween 80, 10 μl/L vitamin K_1_, and 1.0 mg/L resazurin solution.

Fresh stool samples were provided by three healthy volunteers (18–25 years old) who had not taken any antibiotics in the past 3 months. An autoclave was used to sterilize the stool for 20 min at 121°C before use. Stool samples from each donor were mixed in equal amounts and immediately homogenized in sterile phosphate‐buffered saline (0.1 M, pH 7.2) for 1 min to obtain a stool mixture (10% w/v). The final samples were collected after filtration through four layers of sterile gauze. The filtrate was immediately stored in an anaerobic tank.

Next, 1.0 ml of 10% fecal filtrate was added to 9.0 ml of the abovementioned medium as a normal control group (group N), while the same volume of the fecal filtrate was added to 9.0 ml of medium containing 0.1 g of TBP as a TBP group (group T) (to simulate the impact of ingestion of TBP on the growth of GM). The abovementioned procedure was followed by anaerobic fermentation at 37°C. The broth of samples was collected at 0, 6, 12, 24, and 48 h during the fermentation and stored in an ultra‐low‐temperature frozen storage box until use.

The broth samples were collected after 0 and 48 h of the fermentation and their total microbiome DNA was extracted using a DNA extraction kit (ZR Fecal DNA MiniPrep Kit, Zymo Research Corporation) and quantified using a Nanodrop. The quality of extracted DNA was detected by 1.2% agarose gel electrophoresis. PCR was performed with genomic DNA as the template and specific barcoded primers. The forward (5′‐ACTCCTACLGGGALGGCAGCA‐3′) and reverse (3′‐TCLGGACTACHVLGGGTWTCTAAT‐5′) primers were designed and used for the polymerase chain reaction (PCR) amplification of V3–V4 of 16srRNA. The amplified products were recovered, purified, and quantified using tool. According to the fluorescence‐based quantification results, each sample was mixed in the corresponding ratio according to the sequencing volume required for each sample. The sequencing library was prepared using TruSeq Nano DNA LT Library Prep Kit from Illumina and high‐throughput sequencing was performed on the MISEQ Illumina (PE300) sequencing platform.

### 2.7 Changes in the SCFA during fermentation

The method described by Liu et al. (Liu et al., 2022) was used to determine the SCFAs content. Briefly, the fermentation broth was centrifuged and added to the anhydrous ethanol in a 1:1 ratio, vortexed, mixed, and centrifuged. The supernatant was used for gas chromatography–mass spectrometry (GC–MS) analysis. The peak area was recorded, and the corresponding SCFA concentration was calculated using the standard curve. The chromatographic conditions were as follows: chromatographic column: Agilent DB‐WAX and capillary column (30 m × 0.25 mm ID × 0.25 μm); injection volume: 1 μl; inlet temperature: 250°C; ion source temperature: 230°C; transmission line temperature: 250°C; and quadrupole temperature: 150°C. The starting temperature of the programmed ramp‐up was 90°C; it was ramped up to 180°C at a speed of 10°C/min and maintained for 2 min and then ramped up to 250°C at 25°C/min and maintained for 2 min. Nitrogen was used as the carrier gas, with a flow rate of 1.0 ml/min. The MS conditions were as follows: electron impact ionization source, full scan, SIM scan mode, and electron energy of 70 eV.

### 2.8 Statistical analysis

All the experiments were performed in triplicates and the results were expressed as mean ± standard deviation. One‐way analysis of variance was performed using IBM SPSS Statistics for Windows, version 26 (IBM Corp.). Significant differences between the groups were determined by Duncan's post hoc test. The *p* values <.05 were considered statistically significant. Plots were constructed using Origin 2018 (OriginLab Corporation) and GraphPad Prism version 9 for Windows (GraphPad Software).

## 3 RESULTS AND DISCUSSION

### 3.1 Isolation and purification of TBP

Tartary buckwheat has various health promoting effects due to its unique bioactive components, such as flavonoids, phenolic acids, triterpenes, and active polysaccharides (Zou et al., 2021). Currently, the available studies have majorly focused on the active substances (flavonoids, polyphenols, etc.) and biological functions of Tartary buckwheat on the hypoglycemic effects especially blood lipids of flavonoids and polyphenols. However, very few studies highlight the role of other components such as polysaccharides. In this study, four different TBP fractions, including TBP‐W, TBP‐0.2, TBP‐0.5, and TBP‐1.0, were collected. Labeling of the fractions corresponds to the use of water, 0.2, 0.5, and 1.0 M sodium chloride as the eluent resulting in about 0.37%, 2.3%, 4.2%, and 1.7% yield, respectively. The highest content obtained in the fraction TBP‐0.5 was further purified using a Sephadex column to generate two major subfractions. The symmetric fraction at 110–160 min (the first peak) was collected as fraction A, while the symmetric fraction at 210–280 min(the second peak) was collected as fraction B. The polysaccharides TBP‐0.5A and TBP‐0.5B were purified on a gel column with yields of 3.9% and 6.9%, respectively (Figure S1–S4).

### 3.2 Identification of TBP structure

TBP‐W, TBP‐0.2, and TBP‐0.5A were mainly composed of polysaccharides with two relative molecular masses. The molecular weight (Mw) of TBP‐W was 2,515,944 Da at peak 1 and 3667 Da at peak 2. The Mw of TBP‐0.2 was 1,464,817 Da at peak 1 and 16,512 Da at peak 2. The Mw of TBP‐0.5A was 1,271,156 Da at peak 1 and 8559 Da at peak 2. There was only one main peak in TBP‐0.5B and TBP‐1.0. The Mw of TBP‐0.5B was 56,807 Da and that of TBP‐1.0 was 60,408 Da (Table 1).

**TABLE 1:** Molecular weight distribution of TBP

Sample name	Retention time/min	Mp/Da	Mw/Da	Mn/Da	Area/%
TBP‐W	30.991	1,599,519	2,515,944	1,276,646	20.200
	45.166	3494	3667	2987	79.800
TBP‐0.2	32.165	963,007	1,464,817	772,994	23.016
	41.9	14,334	16,512	12,060	76.984
TBP‐0.5A	32.266	836,175	1,271,156	674,967	88.131
	43.207	7694	8559	6565	11.869
TBP‐0.5B	39.066	45,375	56,807	37,911	100
TBP‐1.0	39.085	48,389	60,408	40,163	100

*Note*: TBP‐W, TBP‐0.2, and TBP‐1.0 were isolated by water, 0.2 M NaCl, and 1.0 M NaCl, respectively. TBP‐0.5A and TBP‐0.5B were isolated and purified by Sephadex column with 0.5 M NaCl.

It was observed that *N*‐acetyl‐α‐d‐glucosamine, d‐mannose, d‐ribose, galacturonic acid, and mannuronic acid were not identified in TBP‐W, TBP‐0.2, TBP‐0.5A, TBP‐0.5B, and TBP‐1.0. There were certain differences found in their monosaccharide compositions. While TBP‐W was mainly composed of glucose, d‐galactose, and fructose, TBP‐0.2 was composed of d‐galacturonic acid, d‐galactose, xylose, and arabinose. One the other hand, TBP‐0.5A was composed of d‐galactose and xylose and TBP‐0.5B was composed of glucose and d‐galacturonic acid. TBP‐1.0 was mainly composed of fructose, glucose, and d‐galactose (Table 2).

**TABLE 2:** Molar ratio of TBP‐W, TBP‐0.2, TBP‐0.5A, TBP‐0.5B, and TBP‐1.0

No.	Name	Retention time/min	The molar ratio of				
			TBP‐W	TBP‐0.2	TBP‐0.5A	TBP‐0.5B	TBP‐1.0
1	Fucose	4.8	0.003	0.004	0.008	0	0.269
2	Galactosamine hydrochloride	8.775	0	0.002	0	0	0
3	Rhamnose	9.35	0.036	0.050	0.052	0.187	0.119
4	Arabinose	10.059	0.050	0.118	0.038	0.026	0.054
5	Glucosamine hydrochloride	11.067	0.013	0.015	0	0.019	0.006
6	Galactose	12.667	0.153	0.226	0.425	0.096	0.151
7	Glucose	14.325	0.623	0.087	0.038	0.357	0.241
8	*N*‐Acetyl‐d glucosamine	15.25	0	0	0	0	0
9	Xylose	16.742	0.009	0.150	0.195	0	0.074
10	Mannose	17.3	0.108	0.053	0.099	0	0.034
11	Fructose	19.484	0	0	0	0	0
12	Ribose	21.45	0	0	0	0	0
13	Galacturonic acid	43.917	0.007	0.271	0.078	0.316	0.040
14	Guluronic acid	44.342	0	0	0	0	0
15	Glucuronic acid	46.642	0	0.025	0.067	0	0.012
16	Mannuronic acid	49.109	0	0	0	0	0

*Note*: TBP‐W, TBP‐0.2, and TBP‐1.0 were isolated by water, 0.2 M NaCl, and 1.0 M NaCl, respectively. TBP‐0.5A and TBP‐0.5B were isolated and purified by Sephadex column with 0.5 M NaCl.

### 3.3 Effect of different polysaccharides on the growth of probiotics

**FIGURE 1:**
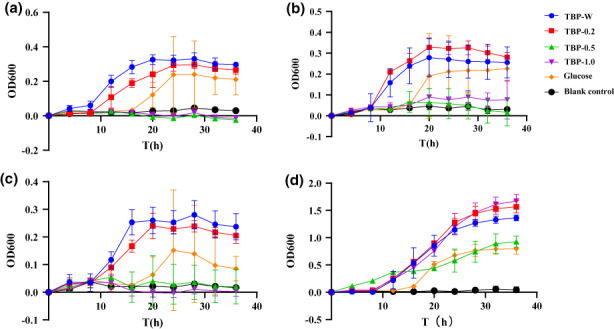
Utilization of different carbon sources by probiotic bacteria. Growth curves of (a) *Bifidobacterium longum*, (b) *Bifidobacterium breve*, (c) *Bifidobacterium adolescentis*, and (d) *Lactobacillus rhamnosus*

*Bifidobacterium* and *Lactobacillus* are well recognized as probiotics and play a vital role in human health such as digestion. The relative abundance of *Bifidobacterium* in the intestine decreases with age. Due to the importance of *Bifidobacteria* for health, there is a growing interest in the maintenance, increase, and recovery of *Bifidobacteria* populations in the human intestine (Kelly et al., 2021). Besides, LGG is the most researched and clinically tested probiotic worldwide. It can be crucial in preventing alcoholic gastric ulcers and regulating immunity (Da et al., 2021; Rongrong et al., 2021). The growth curves of three *Bifidobacterium* species and LGG on TBP‐W, TBP‐0.2, TBP‐0.5, TBP‐1.0, glucose, and MRS medium without glucose are depicted in Figure 1. It can be seen that the growth effect of different TBP components on the four probiotic bacteria are different. The monosaccharide composition of TBP‐W and TBP‐0.2 significantly promoted the proliferation of *B. longum*, *B. breve*, *B. adolescentis*, and LGG. The effect was found to be better than that of glucose. It should be noted that the effects of TBP‐W and TBP‐0.2 on different types of *Bifidobacteria* was not identical. For instance, for *B. longum* and *B. adolescentis*, the added value of TBP‐W was better than that of TBP‐0.2, whereas for *Bifidobacterium*, the added value of TBP‐0.2 was better than that of TBP‐W. The disparity was presumably due to the difference in the transmembrane transport systems involved in the utilization of polysaccharides by each *Bifidobacterium*, their respective glycosidase activities, and metabolic pathways (Wei, 2019). On the other hand, TBP‐0.5 and TBP‐1.0 were barely utilized by *Bifidobacterium*, but could be utilized by LGG with an intensity of action comparable to that of glucose. Consistent with the results shown by Salli et al. (2021), our findings suggest that microbial utilization of sugars is selective, with differences in the carbon source utilization between species and even between strains of the same species. These results also highlight the potential of different plant‐derived polysaccharides to selectively modulate gut microbes. Insights into the mechanisms of action of polysaccharides that can effectively target the proliferation of specific probiotic bacteria may provide opportunities to increase the abundance of probiotics in the human gut through dietary approaches (Kelly et al., 2021) and modulate organismal health.

### 3.4 Effect of TBP on the composition of GM

GM provides complementary genetic resources for energy acquisition, production of essential vitamins, gut maturation, and immune system development to the host (Shin et al., 2015). Dietary intervention is among the main approaches leading to individual microbiota variation (Rothschild et al., 2018) and the easiest factor to modify and control. Polysaccharides, the most abundant dietary component, are crucial substrates for microbial utilization in the distal colon. These compounds can modulate host health by altering the composition of GM. It should be noted that TBP is not digested and absorbed by the small intestine and gets transferred into the large intestine after the consumption of buckwheat. To evaluate the microbial effects of TBP, we analyzed the changes in GM structure during fecal fermentation with TBP (group T, TBP group) versus without TBP (group N, normal control group) through in vitro fermentation experiments using high‐throughput sequencing technology.

The Chao1 and observed species index, which characterized the abundance of fecal microbiota, were significantly higher in group T than in group N (*p* < .01). These findings indicate that TBP enhanced the biodiversity of the microbial community. The Shannon/Simpson index values were close in the two groups as both the values significantly declined (*p* < .01). A decrease in the Shannon and Simpson indices in group T was attributed to the competitive effect of dominant microbiota (Li et al., 2020). Moreover, the diversity was enhanced in group T, where the addition of TBP increased the population of dominant bacteria in the microflora. The addition of TBP enhanced the α and β diversities of microbial communities during in vitro fermentation (Figure S4), suggesting that the carbon source primarily influenced the composition of GM.

**FIGURE 2:**
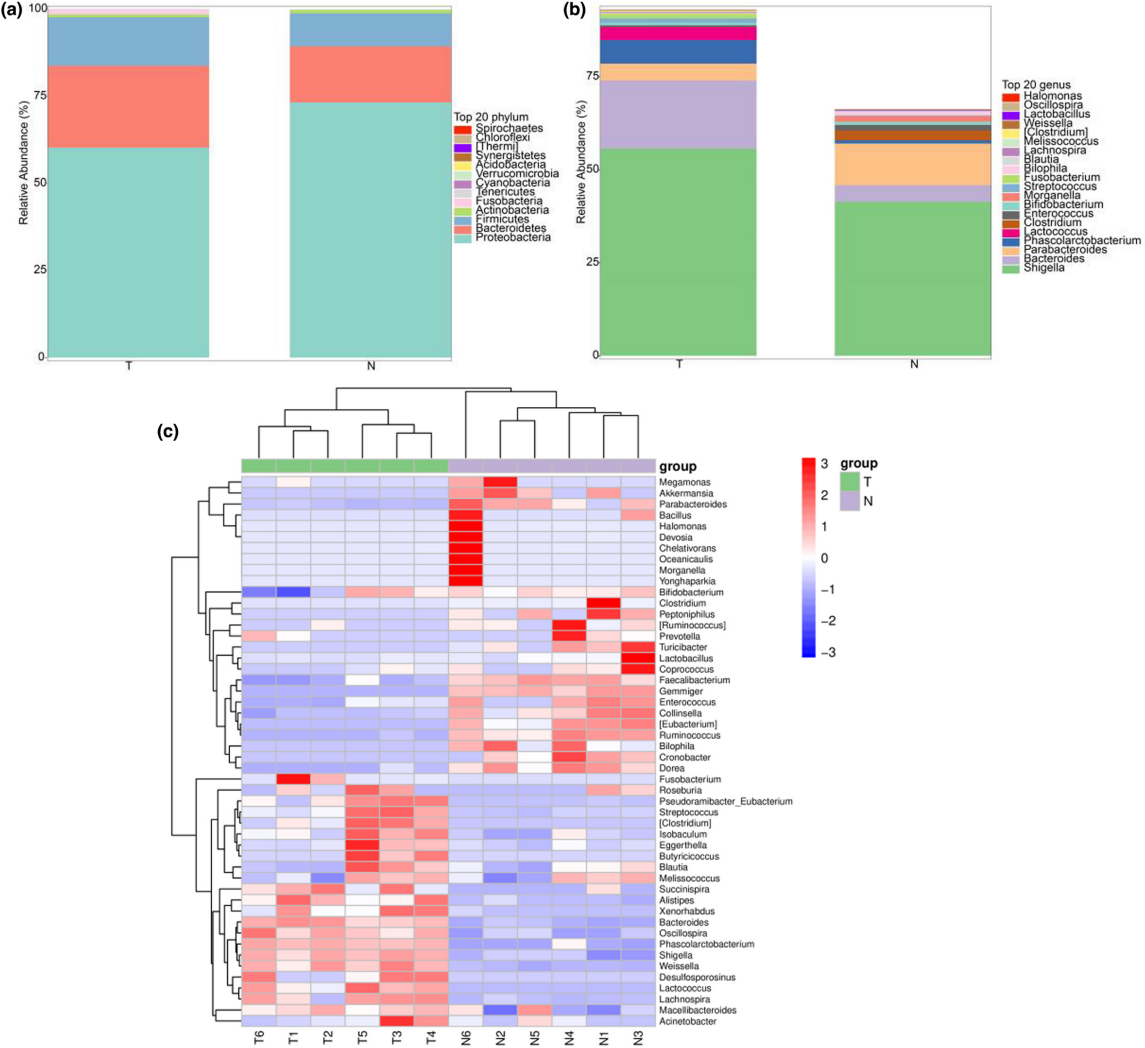
Effects of in vitro fermentation of TBP on GM. The relative abundance of microbial community in (a) phylum and (b) genus. (c) Heat map of species composition at the genus level for biclustering of GM colony structure

The relative abundance of microbiota at the phylum level is shown in Figure 2a. The proteobacteria in group T were significantly less than those in group N (*p* < .05), while the bacteroidetes were markedly higher than those of group N (*p* < .05). The addition of TBP causes a decrease in the normal composition of proteobacteria, as well as an increase in the bacteroidetes and firmicutes. According to the existing studies, proteobacteria are the marker of intestinal microdysbiosis, including various pathogenic bacteria, such as *Escherichia coli*, *Salmonella*, and *Shigella*. An increase in the proteobacteria can lead to nutritional and metabolic disorders, and immune dysregulation in the host (Shin et al., 2015). Therefore, its reduction might be beneficial for host health. Next, the bacteroidetes belong to the major beneficial microbiota that is involved in the fermentation and utilization of polysaccharides to produce SCFAs, especially acetate and propionate (Patnode et al., 2019). The production of SCFAs is dependent on a plethora of discrete polysaccharide utilization loci that are selectively activated to facilitate glycan capture at the cell surface (Foley et al., 2016). Polysaccharides serve as the main source of energy for bacteroidaceae. The bacteroidetes can degrade various plant polysaccharides, thus increasing their relative abundance (Tamura et al., 2017). The previous finding justifies the higher relative abundance of bacteroidetes in group T as compared to group N. Among the other two major phyla, the firmicutes contain a variety of beneficial bacteria, such as *Lactobacillus*, and pathogenic bacteria exerting more complex health effects. In addition, actinobacterium is a common probiotic represented by *Bifidobacterium*. Sequencing results at the phylum level showed that adding TBP promoted the growth of firmicutes, whereas decreased the growth of actinobacteria. However, the difference between the two fermentation groups was insignificant (*p* > .05). For the firmicutes and actinobacteria, both beneficial bacteria and pathogenic bacteria were present. While most members of the firmicutes are beneficial bacteria such as *Lactobacillus*; actinobacteria are often represented by *Bifidobacterium*. Although the existence of pathogenic bacteria *Streptococcus* and *Mycobacterium paratuberculosis* cannot be ignored, it can be perceived that TBP regulates the composition and abundance of GM, which can significantly increase and reduce the relative abundance of bacteroidetes and proteobacteria, respectively.

The corresponding abundance results at the genus level are shown in Figure 2b. The relative abundance of *Shigella* genera, *Bacteroide*s, and *Phascolarctobacterium* was increased significantly (*p* < .05) with the addition of TBP as compared to the control (without TBP addition). It was previously demonstrated that *Phascolarctobacterium* can consume succinate, thereby reducing its amount available in the intestinal lumen to inhibit the growth of *Clostridium difficile* (Nagao‐Kitamoto et al., 2020). Notably, the relative abundance of *Shigella* genera was significantly increased in the TBP group, which is consistent with the findings by Wu et al. (Wu et al., 2021). The possible reason could be the potential of *Shigella* to utilize a low‐molecular‐weight carbon source supporting its growth in the group T. In TBP in vitro fermentation experiments, the relative abundance of *Parabacteroides distasonis* was markedly reduced in the group T relative to the control group (*p* < .05). It has been found that higher levels of *P. distasonis* are associated with more pronounced memory deficits (Noble et al., 2021). Generally, TBP promotes the growth of beneficial bacteria and inhibits harmful bacteria. However, a simultaneous increase in the growth of harmful bacteria *Shigella* genera cannot be ignored. Therefore, in‐depth studies on the effect of polysaccharides on the structure of the GM are required as a future goal. To further compare the species composition differences among samples and demonstrate species abundance distribution trend for each sample, a heat map was generated for the composition analysis of different species. Figure 2c reveals the abundance data of the top 50 genera in terms of mean abundance in groups T and N. In both groups, the relative abundance of beneficial genera, including *Bacteroides* and *Phascolarctobacterium* increased, while the relative abundance of harmful genera, such as *Bilophila* and *Cronobacter* spp., decreased. During the composition analysis of GM, we also focused on the gut probiotics that were found to be relatively abundant in our current research. In particular, *Bifidobacterium* can regulate gastrointestinal function, promote gastrointestinal health, and strengthen intestinal immunity (Ding et al., 2019), and *Lactococcus* which could control serum cholesterol level, regulate the immune system, and has anticancer activity (Di Gioia et al., 2014). However, their relative abundances were very low, which might be related to the culture conditions. The cluster dendrogram reflected the similarity of bacterial composition, and the specificity of bacterial distribution. Cluster analysis by the bacterial community showed that the bacterial community of group N exhibits a distant relationship with the bacterial community of group T. The alteration of TBP for GM was more significant. Thus, it can be concluded that TBP supplementation altered the microbial composition by inhibiting the growth of harmful bacteria and promoting the growth of beneficial bacteria.

### 3.5 Effect of TBP on the composition of SCFAs

SCFAs are the main metabolites produced after fermentation and play a crucial role in immunity, inflammation, and metabolism (Yao et al., 2022). The concentration of SCFAs is often used as an indicator to determine the fermentability of polysaccharides. In this study, GC/MS was used to determine the changes in the SCFAs composition during the fermentation process. Given that the levels of caproic, valeric, isobutyric, and isovaleric acids were low and remained significantly unchanged during fermentation, only three major SCFAs, namely acetic acid, propionic acid, and butyric acid, were analyzed.

GM can utilize TBP to produce SCFAs (Table 3). Notably, the experimental group with added TBP had significantly higher levels of acetic acid, propionic acid, and butyric acid than the control group during 0–48 h fermentation. Acetic acid increased from 18.17 to 65.88 μg/ml, while the level of propionic acid enhanced from 11.78 to 38.74 μg/ml. These results are in agreement with the previous studies on the fermentation of okra polysaccharides among other polysaccharides in the fecal flora (Wu et al., 2021). Acetic acid has been shown as a source of energy for the GM (with the most abundant SCFA in the peripheral circulation) to cross the blood–brain barrier. It is metabolized in the muscle, kidney, heart, and brain to provide energy (Oliveira et al., 2012). Bartolomaeus et al. (2018) reported that propionic acid prevented the damage of target organs in hypertensive and atherosclerotic mice. The mechanism was mediated by maintaining the immune homeostasis against hypertension‐induced cardiovascular function impairment, thereby playing a crucial role in cardiovascular health. In particular, butyric acid is useful in improving health. It has multiple physiological effects, such as maintaining intestinal homeostasis by regulating synaptopodin (SYNPO) expression. Therefore, butyric acid promotes intestinal barrier function and accelerates the repair of intestinal epithelial cell damage (Wang et al., 2020). Taken together, TBP can be degraded and utilized by the GM during in vitro fermentation to produce a variety of SCFAs that are beneficial to human health.

**TABLE 3:** The concentration of short‐chain fatty acids (SCFAs) at different time points of fermentation

Group	Time (h)	SCFA(μg/ml)		
		Acetic acid	Propionic acid	Butyric acid
*T*	0	18.17 ± 0.53f	11.78 ± 0.04c	16.49 ± 0.07b
	6	20.32 ± 0.81ef	11.81 ± 0.04c	16.49 ± 0.02b
	12	32.00 ± 1.98c	12.59 ± 0.08c	16.65 ± 0.06b
	24	44.60 ± 3.49b	13.29 ± 1.11c	17.08 ± 1.08b
	48	75.88 ± 2.67a	45.41 ± 2.68a	26.78 ± 1.13a
*N*	0	22.10 ± 3.28e	12.88 ± 0.56c	16.63 ± 0.20b
	6	21.28 ± 1.09ef	12.52 ± 0.16c	16.43 ± 0.08b
	12	22.60 ± 0.48e	12.26 ± 0.29c	16.44 ± 0.04b
	24	22.92 ± 0.07e	12.26 ± 0.03c	16.30 ± 0.03b
	48	27.18 ± 1.05d	15.28 ± 0.20b	16.68 ± 0.06b

*Note*: *T*, the experimental group (TBP supplement); *N*, the control group (no additional carbon source supplement). The results are expressed as the mean ± standard deviation, and different letters in the same column are significant (*p* < .05).

## 4 CONCLUSION

The current study revealed that four different structural polysaccharide fractions of TBP efficiently affected the proliferation of specific probiotic bacteria (*B. longum*, *B. breve*, *B. adolescentis*, and LGG). The growth of microbiota was found to be associated with the molecular weight and monosaccharide composition of different fractions. We observed that the TBP was significantly degraded and utilized by the human GM. As a result, it increased the production of beneficial microorganisms and inhibited the growth of harmful microorganisms. Furthermore, it promoted the production of acetic acid, propionic acid, and butyric acid. We postulate that TBP can change the composition and abundance of human GM, improve the intestinal microenvironment, and ultimately offer several benefits to human health. Taken together, the current study suggests that TBP has probiotic properties and can reshape the GM composition to exert physiological effects. Our findings further suggest that the intake of different sources of plant polysaccharides may be important in modulating intestinal microecology.

## CONFLICT OF INTEREST

All authors disclosed no relevant relationships.

## Supporting information


Figure S1–S4

## Data Availability

Data available on request from the authors.
