# Supplementary material for: Isolation and purification of Tartary buckwheat polysaccharides and their effect on gut microbiota
Source: Food Sci Nutr. 2022 Sep 27;11(1):408–17. doi: 10.1002/fsn3.3072 (PMC9834889; doi:10.1002/fsn3.3072)
Supplement: Supplementary file 1 — Figure S1–S4 [file FSN3-11-408-s001.docx]

Figure S1. Separation and purification of TBP. **A**, Elution curve of crude TBP on DEAE Sepharose Fast Flow column. **B**, Elution curve of TBP-0.5 on Sephadex.


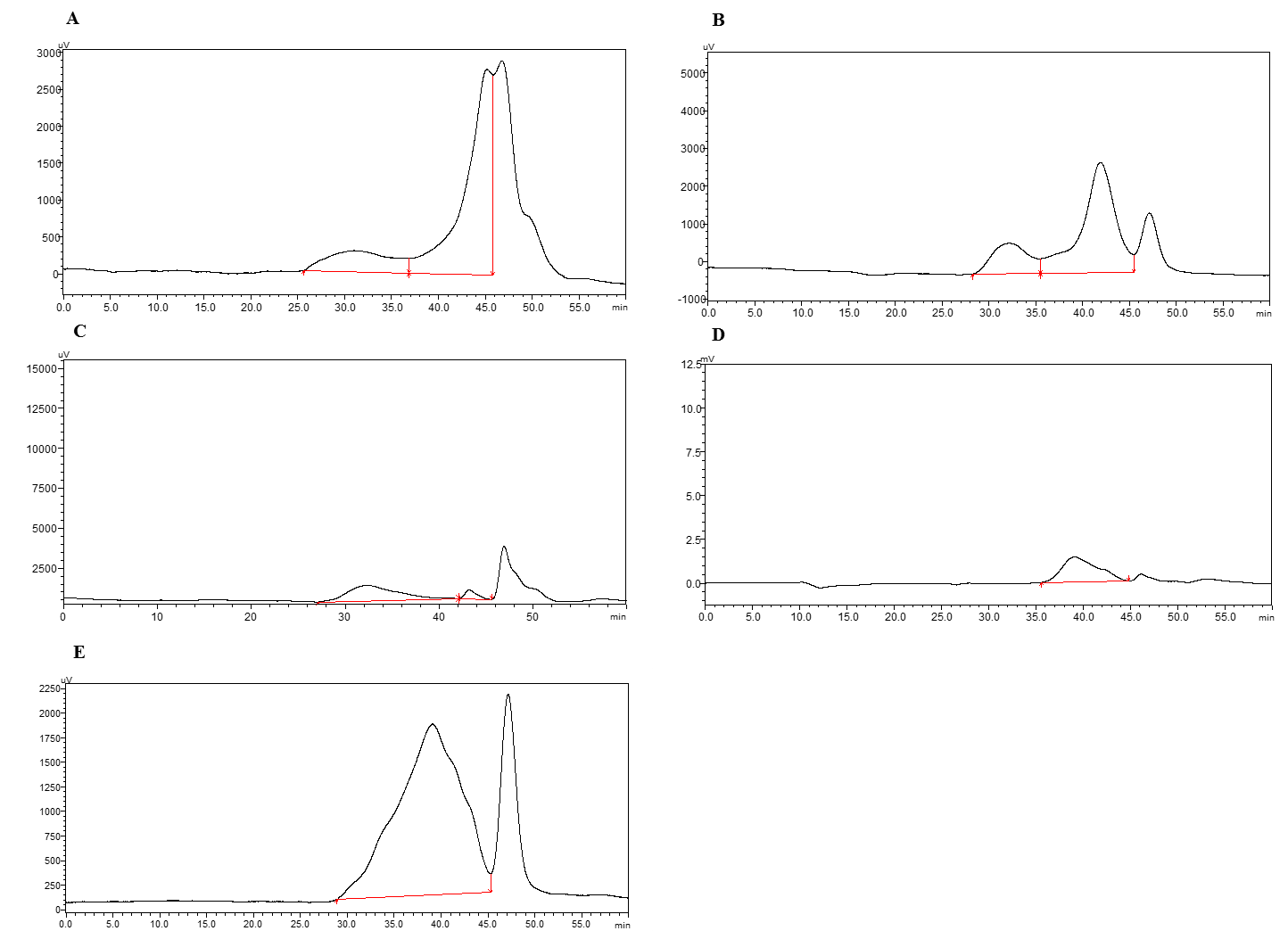


Figure S2. Molecular weight determination of TBP. **A-E**, The HPGPC chromatogram of TPP-W, TBP-0.2, TBP-0.5A, TBP-0.5B and TBP-1.0 respectively. Around 46.8min is the peak of the mobile phase.

Figure S3. Monosaccharide composition determination of TBP. A-F, IC chromatogram of Standard Sugar, TBP-W, TBP-0.2, TBP-0.5A, TBP-0.5B and TBP-1.0 respectively. The numbers represent different monosaccharides. **1**, Fucose; **2**,Galactosamine hydrochloride; **3**, Rhamnose; **4**, Arabinose; **5**, Glucosamine hydrochloride; **6**, Galactose; **7**, Glucose; **8**, N-acetyl-D glucosamine; **9**, Xylose; **10**, Mannose; **11**, Fructose; **12**, Ribose; **13**, Galacturonic acid; **14**, Guluronic acid; **15**, Glucuronic acid; **16**, Mannuronic acid.

Figure S4. Effects of TBP on GM diversity after fecal fermentation for 48 h. **A**, Grouped box plot of Alpha Diversity Index. **B**, Venn diagram for OTUs in the group T and group N. **C**, PCA analysis for microbial beta-diversity in groups T and N. The significance is indicated by *P < 0.05 and **P < 0.01.
